# Grooming Up the Hierarchy: The Exchange of Grooming and Rank-Related Benefits in a New World Primate

**DOI:** 10.1371/journal.pone.0036641

**Published:** 2012-05-08

**Authors:** Barbara Tiddi, Filippo Aureli, Gabriele Schino

**Affiliations:** 1 Research Centre in Evolutionary Anthropology and Palaeoecology, School of Natural Sciences and Psychology, Liverpool John Moores University, United Kingdom; 2 Cognitive Ethology Laboratory, German Primate Center, Goettingen, Germany; 3 Instituto de Neuroetologia, Universidad Veracruzana, Xalapa, México; 4 Istituto di Scienze e Tecnologie della Cognizione, C.N.R, Rome, Italy; Texas A&M University, United States of America

## Abstract

Seyfarth's model assumes that female primates derive rank-related benefits from higher-ranking females in exchange for grooming. As a consequence, the model predicts females prefer high-ranking females as grooming partners and compete for the opportunity to groom them. Therefore, allogrooming is expected to be directed up the dominance hierarchy and to occur more often between females with adjacent ranks. Although data from Old World primates generally support the model, studies on the relation between grooming and dominance rank in the New World genus *Cebus* have found conflicting results, showing considerable variability across groups and species. In this study, we investigated the pattern of grooming in wild tufted capuchin females (*Cebus apella nigritus)* in Iguazú National Park, Argentina by testing both the assumption (i.e., that females gain rank-related return benefits from grooming) and predictions (i.e., that females direct grooming up the dominance hierarchy and the majority of grooming occurs between females with adjacent ranks) of Seyfarth's model. Study subjects were 9 adult females belonging to a single group. Results showed that grooming was given in return for tolerance during naturally occurring feeding, a benefit that higher-ranking females can more easily grant. Female grooming was directed up the hierarchy and was given more often to partners with similar rank. These findings provide supporting evidence for both the assumption and predictions of Seyfarth's model and represent, more generally, the first evidence of reciprocal behavioural interchanges driven by rank-related benefits in New World female primates.

## Introduction

The distribution of grooming among group members is a fundamental aspect of primate sociality with direct implications for social bonding. In addition to its hygienic function [1], contributions from different research fields have promoted the view of grooming as a social tool that facilitates bonding between individuals through neuropeptide-based pain-control mechanisms (see Dunbar for a recent review [2]). For example, the pharmacological blockade of brain opioid receptors increases the need for social comfort and thus the requests for grooming [3], [4]; similarly, receiving grooming increases the natural release of brain opioids [5]. In addition, recent studies have provided compelling evidence that social bonds maintained through long-term grooming interactions enhance individual fitness in both female [6], [7], [8] and male primates [9].

Grooming is considered to be a low-cost service that individuals can exchange for other kinds of benefits [10], [11]. While some of these return benefits (e.g., additional grooming) can be provided by any group member, others (e.g., agonistic support) are more easily offered by high-ranking individuals. If grooming is exchanged for benefits best provided by high-ranking individuals, this is likely to affect how animals distribute their grooming among group members. This was first noted by Seyfarth [12], who proposed a now influential model to explain grooming patterns among female primates. In this model, grooming is assumed to be offered in return for benefits best provided by the highest-ranking females, such as tolerance over food resources and agonistic support during conflicts. In addition, by considering time available for grooming as a limiting factor on social interactions [13], Seyfarth's model predicts that females compete for the opportunity to groom higher-ranking females. Here, assuming that high-ranking females experience the least competition for preferred partners, they are free to more frequently groom other high-ranking individuals. In contrast, middle-ranking females have fewer opportunities to access higher-ranking grooming partners because they are out-competed by higher-ranking females; middle-ranking females thus direct most of their grooming to other middle-ranking females. For the same reason, low-ranking females are primarily limited to grooming other low-ranking individuals. The end result of these processes is that females direct their grooming up the dominance hierarchy and that most grooming occurs between females of adjacent ranks [13], [14].

Studies of Old World primates have provided consistent evidence that females direct their grooming up the dominance hierarchy [15]. However, much more controversial is the underlying assumption that females groom others in return for rank-related benefits, particularly agonistic support. For instance, evidence of reciprocal interchanges between grooming and support was found in Japanese macaques (*Macaca fuscata*) [16], but not in chacma baboons (*Papio hamadryas ursinus*) [17]. Such inconsistency has been partly superseded by a recent meta-analysis including 14 different primate species [18], in which a weak but highly significant correlation between grooming and support was found.

Criticism of Seyfarth's [12] model has stemmed from the view that, given the rarity of agonistic support [19], [20], grooming is unlikely to be interchanged for such an uncertain future benefit. However, grooming may be interchanged with commodities other than support [19], [20]. Indeed, there is evidence that grooming is associated with other rank-related benefits, such as a reduction of received aggression [21] and an increase in the access to clumped, contestable resources [22], [23]. However, efforts aimed at testing Seyfarth's model have remained mostly focused on the interchange between grooming and agonistic support, with only a few studies investigating other forms of rank-related benefits [22], [23], [24], [25].

**Figure 1:**
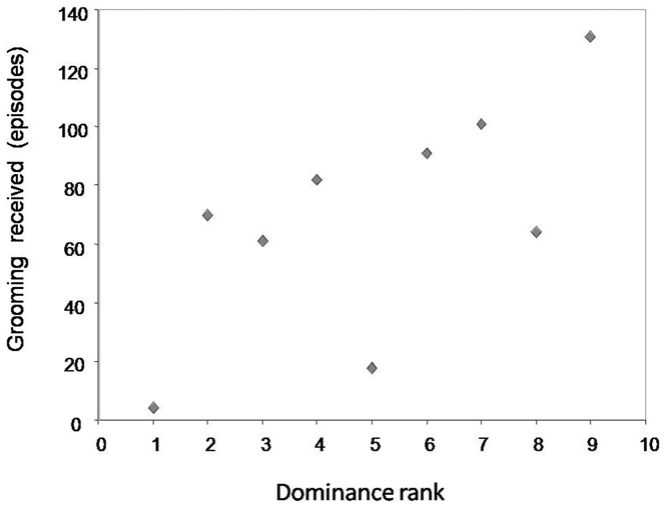
Grooming received in relation to the receiver's dominance rank. Higher ranks are indicated by higher numerical values.

This study investigated the female-female grooming patterns in a large group of wild tufted capuchin monkeys (*Cebus apella nigritus*, taxonomically synonymous with *Sapajus nigritus*) with the aim of testing Seyfarth's model. In particular, this study represents the first attempt in wild New World primates generally, and in the genus *Cebus* specifically, to analyse grooming distribution in relation to multiple rank-related benefits, namely agonistic support, reduced aggression, and tolerance during feeding. Previous investigations testing the predictions of Seyfarth's model [12] regarding grooming distribution in the genus *Cebus* have yielded conflicting results. Perry [26] suggested that *C. capucinus* females groom up the hierarchy following the predictions of Seyfarth's model, whereas the opposite was found in *C. olivaceus* [27] and *C. apella* [28]. Although these analyses tested the predictions of Seyfarth's model by detailing the distribution of grooming among females in relation to dominance rank, few studies focused on verifying the assumption of the model (i.e., that grooming increases the likelihood of gaining rank-related return benefits; but see [26], [29]). Therefore, questions about whether or not females gain benefits by grooming dominant individuals, what kind of benefits are received, and the consequences of such exchanges are still open.

The present study aimed at testing both the assumption and predictions of Seyfarth's model. We tested the assumption that tufted capuchin females gained rank-related benefits in return for grooming by examining whether grooming was associated with: a) the increased likelihood of receiving agonistic support (i.e., whether females supported more often those females that groomed them most); b) the reduced likelihood of receiving aggression (i.e., whether females were less often aggressive against those females that groomed them most); c) the increased likelihood of receiving tolerance at food sources (i.e., whether females tolerated preferentially those females that groomed them most). Among the predictions of Seyfarth's model, we tested whether tufted capuchin females directed grooming up the dominance hierarchy, that is whether females gave more grooming to higher- rather than lower-ranking females (Prediction 1); and higher-ranking females received overall more grooming than lower-ranking females (Prediction 2). Then, we tested if competition for preferred grooming partners occurs by examining whether tufted capuchin females directed the majority of their grooming to females of adjacent ranks (Prediction 3); whether higher-ranking females were better able than lower-ranking females to allocate their grooming according to the rank of the recipient (Prediction 4); and whether higher-ranking females performed overall more grooming than lower-ranking females (because grooming allocation in the latter was constrained by dominance relations) (Prediction 5).

## Materials and Methods

### (a) Ethics statement

This study was conducted in accordance with the Animal Behaviour Society's guidelines for the treatment of animals in behavioural research and teaching. In addition, permission to conduct research was provided by the Administración de Parques Nacionales in Argentina (no permission ID was given). Data collection was entirely based on observations of fully-habituated, wild groups and did not affect the monkeys' welfare. Because the study was only observational and approved by the local authorities, our institutions did not require an application to the ethics committee.

### (b) Study subjects

Data were collected between June 2006 and March 2007 in Iguazú National Park, Argentina (25°40′S, 54°30′W). The park is located in the northwestern side of the waterfalls of the Iguazú River and is characterized by semi-deciduous forest with a humid sub-tropical climate and marked seasonality in daylight duration and temperature [30].

Tufted capuchin monkeys are highly arboreal, medium-sized (2.5–3.6 kg; [31]) New World primates. Tufted capuchins are largely frugivorous, although a considerable portion of their diet consists of insect prey [32]. Study subjects were 9 adult and subadult female capuchins (i.e., ≥4 year-old, the age of the first ovulation; [33]) in a fully-habituated group (the Macuco group). Although female dispersal has been reported to some extent in other populations of this species [34], this study population is characterized by multimale-multifemale groups and female philopatry [35]. Because the group has been the subject of continuous investigation since 1991 [36], maternal kin relationships for females were known [37].

### (c) Data collection

The study group was followed for at least 8 hours per day for up to 25 days per month. Data collection on adult females was based on focal animal sampling, *ad libitum* sampling and scan sampling [38]. During 5-minute focal animal samples, instances of feeding within 3 m from another study subject were scored every minute, recording the individual identities via instantaneous sampling. A 3-meter distance between the focal subject and other individuals in the vicinity was chosen as a compromise between the maximum distance of food monopolization by dominants (i.e., approximately 10 m: [39]) and the need for good visibility through the dense vegetation. Focal females were chosen based on a random permutation schedule with at least 30 minutes separating samples of the same individual. A concerted effort was made to equalize the amount of observations for each female (total focal observation time varying across females from approximately 7 to 11 hours). Scan samples were conducted every 30 minutes throughout the daily observation period to record whether each visible female subject was foraging (i.e., searching for food items on the immediate substrate). All observed episodes of grooming and aggression (i.e., threats, supplants, chases and physical assaults) involving female subjects were scored *ad libitum*. Specifically, grooming was recorded noting the timing and duration of all the episodes as well as the identities of the individuals involved. Aggressive interactions could be dyadic or polyadic (in which multiple individuals were involved). For each polyadic aggressive interaction, only the initial aggressor, the initial receiver and the initial supporter were considered, as it is often impossible to determine the beneficiary of support when many individuals are involved. Agonistic support was considered only if it occurred within 30 seconds from the initial aggressive interaction. The supporter could intervene in the ongoing aggressive interaction aiding either the recipient (i.e., victim support) or the initiator of the original aggression (i.e., aggressor support).

**Table 1:** Test of Seyfarth's model predictions: attraction to and competition for high-ranking females.

Independent variables	β-coefficient	*t*-value	*P*-value
Rank of receiver	0.482	2.80	0.008
Rank distance	−0.394	−2.17	0.034
Kinship	2.041	3.52	0.001

Within-subject linear regression testing whether grooming given (dependent variable) was associated with the rank of the receiver, the rank distance between actor and receiver, and kinship between actor and receiver (N = 72 dyads; df = 60 in all tests).

### (d) Data analysis

**Dominance ranks and calculation of dyadic scores:** Females in the study group were placed in a linear dominance hierarchy based on the direction of dyadic aggressive and approach-avoidance interactions using MatMan 1.1 (Noldus Information Technology 2003; [40]).

Directional dyadic scores of female-female interactions took into consideration the direction of the interaction exchanged between two partners, A and B. Thus, each dyad had two directional dyadic scores for each behaviour: A giving to B, and B giving to A. Directional dyadic scores were calculated considering either all female-female dyads (for grooming and agonistic support) or only dominant-subordinate dyads (i.e., half of the directional dyadic scores; for tolerance during feeding and aggression). Such a distinction was needed because of the obvious influence of female-female dominance relationships on the distribution of tolerance and aggression (i.e., a subordinate female cannot “tolerate” a nearby-feeding dominant female).

Considering a generic dyad A–B, directional dyadic grooming scores of A to B were the total number of grooming episodes given by A to B. Directional dyadic agonistic support scores of A to B were calculated as the number of support cases by A to B divided by the number of opportunities for support, which in turn was the number of aggressive interactions involving B, either as aggressor or as receiver, excluding aggressive interactions between A and B. These scores of agonistic support included all observed episodes of support between two female subjects against any other group member.

Feeding within 3 m from a lower-ranking female was considered as a measure of tolerance by the higher-ranking partner. Therefore, only dominant-subordinate (but not subordinate-dominant) dyads were considered for calculations of directional dyadic scores of tolerance during feeding. If A was dominant over B, dyadic scores of tolerance provided during feeding were calculated as the number of times A was observed feeding within 3 m from B during 1-minute instantaneous samples in focal samples of A and B, divided by the total number of instantaneous samples collected during focal samples of A and B. This value was subsequently divided by an estimate of the proportion of time individual A spent feeding (i.e., the number of 30-minute group scans A was observed foraging divided by the number of group scans in which A was visible), allowing for an estimate of the probability of tolerance corrected for the opportunities A had of tolerating B (i.e., the time A spent foraging). Similarly, directional dyadic scores of aggression were calculated considering dominant-subordinate dyads. If A was dominant over B, directional dyadic aggression scores were the total number of aggressive interactions initiated by A against B.

Finally, time spent in proximity by the dyad A–B was calculated as the number of instances A and B were scored in 3 m proximity during 1-minute instantaneous samples divided by the total number of instantaneous samples collected in focal samples of A and B.

Data points included in the analyses were either all female-female directional dyadic scores (N = 72), or the dominant-subordinate dyadic scores (N = 36) when analyses included tolerance and aggression.

**Testing the assumption of Seyfarth's model:** In order to test whether the likelihood of giving support, tolerance during feeding and reduced aggression were associated with grooming received across female-female dyads, three within-subject linear regressions with robust standard errors [41] were run entering dyadic scores of support, tolerance and aggression given as dependent variables and dyadic scores of grooming received and kinship (the values of the relatedness coefficient *“r”*) as independent fixed effect variables. In order to test the effect of grooming on the three rank-related benefits simultaneously, we also ran a multivariate analysis of covariance (MANCOVA) in which dyadic scores of support, tolerance and aggression given were the dependent variables and dyadic scores of grooming received and kinship were the relevant independent variables. Subject identity was also added as an independent variable in order to obtain a within-subject analysis. Tests of significance were based on Wilk's lambda.

**Testing predictions of Seyfarth's model:** A within-subject linear regression with robust standard errors [41] was run with grooming episodes given as the dependent variable and recipient's dominance rank, rank distance (calculated as the absolute value of the difference in rank between groomer and recipient) and kinship as independent fixed effect variables (Prediction 1 and 3). In addition, Kendall correlation coefficients were used to evaluate the effect of dominance rank on the total amount of grooming received (Prediction 2) and given (Prediction 5) by females.

To test whether higher-ranking females were better able to allocate their grooming in relation to the rank of the recipient (Prediction 4), for each female a Kendall correlation test between the dyadic grooming score and the rank of the grooming recipient was run. The resulting correlation coefficient for each female was then used as a measure of her distribution of grooming in relation to the rank of the recipient; we then tested the relation between these coefficients and the females' own rank with a Kendall correlation test.

As our statistical analyses on grooming combined data collected via *ad libitum* and focal sampling, we assessed the potential occurrence of systematic biases by running a linear regression with grooming data recorded *ad libitum* as dependent variable and grooming data recorded using focal sampling as independent variable. The two variables were strongly correlated (*β* = 4.07, *z* = 11.44, *R^2^* = 0.67, *p*<0.001) confirming that *ad libitum* data provided an unbiased estimate of focal data. All statistical analyses were two-tailed and were run using Stata 10.1 (Stata Corp. 2007).

## Results

### (a) The assumption of Seyfarth's model

Agonistic support given was not associated with the amount of grooming received (*β* = −0.0002, *t* = −0.11, *df* = 61, *p* = 0.911), but females preferentially supported their kin (*β* = 0.025, *t* = 2.36, *df* = 61, *p* = 0.022). Thus, females did not preferentially support those females that groomed them most. Likewise, aggression given was not associated with grooming received (*β* = −0.012, *t* = −0.15, *df* = 24, *p* = 0.882), nor did kinship have a significant relationship with aggression given (kinship: *β* = −0.134, *t* = −0.23, *df* = 24, *p* = 0.819). Adding time spent in proximity as a further independent variable in this analysis (to control for the opportunities dominants had to behave aggressively) did not change the results (aggression: *β* = −0.093, *t* = −1.02, *df* = 24, *p* = 0.314; kinship: *β* = 0.082, *t* = 0.23, *df* = 24, *p* = 0.820; time spent in proximity: *β* = −0.058, *t* = −0.72, *df* = 24, *p* = 0.475). Therefore, dominant females did not refrain from aggressing those subordinate females that groomed them most. By contrast, tolerance given during feeding was positively associated with the amount of grooming received (*β* = 0.02, *t* = 3.35, *df* = 24, *p* = 0.003), and kinship had no significant effect (*β* = 0.06, *t* = 1.10, *df* = 24, *p* = 0.281). Dominant tufted capuchin females thus more often tolerated those subordinate females that groomed them most.

**Figure 2:**
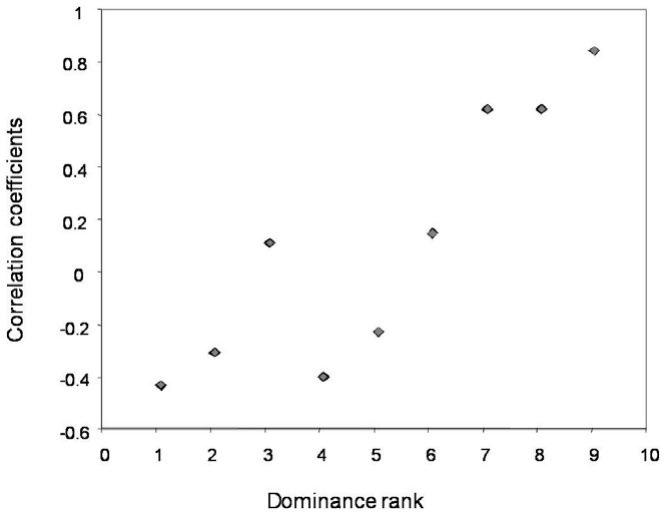
Relation between female dominance rank and ability to groom in relation to the recipient's rank (i.e. correlation coefficients obtained for each female by correlating her grooming with the recipient's rank.) Higher ranks are indicated by higher numerical values.

When the three rank-related benefits were analysed simultaneously in a MANCOVA, they resulted to be significantly related both to grooming received (*Λ* = 0.698, *F* = 3.46, *df* = 3,24, *p* = 0.032) and to kinship (*Λ* = 0.714, *F* = 3.20, *df* = 3,24, *p* = 0.041).

**Predictions of Seyfarth's model**: Grooming given was positively associated with the receiver's dominance rank (Table 1). Thus, tufted capuchin females preferred to groom higher-ranking individuals (Prediction 1). A positive association between total grooming received and dominance rank showed higher-ranking females received overall more grooming than lower-ranking females (Kendall correlation: τ = 0.555, N = 9, p = 0.037; Figure 1; Prediction 2).

**Figure 3:**
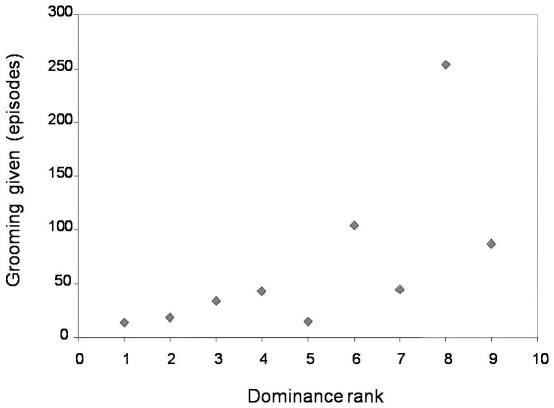
Grooming given in relation to the groomer's dominance rank. Higher ranks are indicated by higher numerical values.

A negative relation between grooming given and rank distance between grooming partners was found (Table 1), suggesting that more grooming was given to females with more similar ranks in the dominance hierarchy (Prediction 3). Higher-ranking females were better able than lower-ranking females to allocate their grooming in relation to the rank of the recipient (Kendall correlation: τ = 0.833, N = 9, p = 0.001; Figure 2; Prediction 4). Additionally, total grooming given was positively correlated with the groomer's dominance rank, indicating that higher-ranking females groomed other females more than lower-ranking females did (Kendall correlation: τ = 0.666, N = 9, p = 0.012; Figure 3; Prediction 5).

## Discussion

The results of this study indicate that inter-individual variation in the distribution of grooming among wild female tufted capuchin monkeys is affected by both dominance rank and the potential for exchanges of grooming for rank-related benefits, thus supporting both the assumption and predictions of Seyfarth's model [12]. In accordance with the model's assumption, capuchin females derived rank-related benefits from grooming higher-ranking females in terms of increased tolerance during feeding. Indeed, dominant females preferentially tolerated those females that groomed them most. Supporting the model's predictions [12], capuchin females preferentially directed their grooming up the dominance hierarchy and appeared to compete for access to higher-ranking females as preferred grooming partners.

Higher-ranking females, however, did not appear to provide agonistic support or to reduce aggression in return for grooming. A possible explanation for these results derives from the special role dominant males exert on group social dynamics in capuchin monkeys. Dominant males appear to have a central position in spatial proximity networks, with females competing for gaining social access to them [42]. Previous studies on tufted capuchin social structure have reported a rather despotic alpha male that aggressively affects the spatial position of group members during feeding and monopolizes a large number of mating opportunities [43], [44], [45]. It is therefore possible that, because females obtain agonistic support from the alpha male, they do not need to trade grooming among themselves for agonistic support.

The published reports of female grooming patterns in the genus *Cebus* shows considerable variability (see Introduction). The reasons for these inconsistencies are unclear, but several explanations are possible. First, in most studies of capuchin monkeys, kinship was unknown and it was thus impossible to control for such a factor. The results of this study show that kinship exerted a profound influence on behaviour: significant effects of kinship were observed on the distribution of agonistic support and grooming. Similarly, Perry et al. [46] showed that kin-biased social behaviour depends on group size and mean relatedness. It seems, therefore, crucial to include kinship data when examining the distribution of capuchin monkeys' social behaviour. In our study, the effect of kinship was controlled for in all relevant analyses, and the evidence supporting Seyfarth's model could not therefore be a by-product of kinship. Second, the variable results obtained thus far might depend on the relatively small group sizes typical of capuchin species, as small sample sizes inevitably lead to more variable results. Third, the distribution of food resources in terms of abundance and patch size might affect the degree of competition and cooperation among group members and in turn alter the nature of rank-related benefits that can be exchanged for grooming [47]. It is thus important to test the assumption of Seyfarth's model, and not only its predictions. Females may show different tendencies to groom up the hierarchy according to variation in the steepness of the dominance gradient, and thus exhibit different patterns in the interchange with grooming (i.e., grooming for grooming when the dominance gradient is shallow and grooming for rank-related benefits when dominance is steep; [48], [49]). Indeed, this pattern of within-species variation was found in a comparative analysis of 38 social groups belonging to 16 primate species [50]. Finally, it is possible that dominance affects capuchin females' sociality to a lesser extent than indicated in many Old World primates [51]. Indeed, dominance relationships appear to be less rigidly enforced and rates of aggression are typically lower in capuchin monkeys than in most macaques, baboons and vervet monkeys [52], [53].

The present study provides the first clear evidence for the interchange of grooming for rank-related benefits (i.e., tolerance during feeding) in wild New World monkeys. Thus far, analyses of the interchange of grooming for rank-related benefits in primates have focused mostly on agonistic support. In this study, we tested the assumption of Seyfarth's model by examining the relation between grooming and multiple forms of rank-related benefits. Our findings suggest that investigating other forms of rank-related benefits may prove fruitful because it is only when the assumption of the model are met that its predictions are expected to hold [27].

In conclusion, even though the relationship between grooming distribution and the acquisition of rank-related benefits warrants further consideration, results from our study highlight two important aspects. First, when testing the social function of grooming, different forms of return benefits should be considered [20]. Second, models initially designed for explaining grooming distribution in Old World primates may have a broader applicability than previously thought (but see [28]). In particular, Seyfarth's predictions on attraction to dominant individuals and competition among partners may successfully explain grooming patterns in New World primates as well, although the kinds of benefits granted by dominant females may differ.
